# Heterogeneous Genetic Background of the Association of Pheochromocytoma/Paraganglioma and Pituitary Adenoma: Results From a Large Patient Cohort

**DOI:** 10.1210/jc.2014-3399

**Published:** 2014-12-12

**Authors:** Judit Dénes, Francesca Swords, Eleanor Rattenberry, Karen Stals, Martina Owens, Treena Cranston, Paraskevi Xekouki, Linda Moran, Ajith Kumar, Christopher Wassif, Naomi Fersht, Stephanie E. Baldeweg, Damian Morris, Stafford Lightman, Amar Agha, Aled Rees, Joan Grieve, Michael Powell, Cesar Luiz Boguszewski, Pinaki Dutta, Rajesh V. Thakker, Umasuthan Srirangalingam, Chris J. Thompson, Maralyn Druce, Claire Higham, Julian Davis, Rosalind Eeles, Mark Stevenson, Brendan O'Sullivan, Phillipe Taniere, Kassiani Skordilis, Plamena Gabrovska, Anne Barlier, Susan M. Webb, Anna Aulinas, William M. Drake, John S. Bevan, Cristina Preda, Nadezhda Dalantaeva, Antônio Ribeiro-Oliveira, Isabel Tena Garcia, Galina Yordanova, Violeta Iotova, Jane Evanson, Ashley B. Grossman, Jacqueline Trouillas, Sian Ellard, Constantine A. Stratakis, Eamonn R. Maher, Federico Roncaroli, Márta Korbonits

**Affiliations:** Department of Endocrinology (J.D., U.S., M.D., P.G., W.M.D., M.K.), Barts and the London School of Medicine, Queen Mary University of London, London EC1M 6BQ, United Kingdom; Semmelweis University, School of PhD studies, Doctoral School of Clinical Medicine, Budapest, Hungary (J.D.), Endocrinology Directorate (F.S.), Norfolk and Norwich University Hospital, Norwich NR4 7UZ, United Kingdom; Department of Medical and Molecular Genetics (E.R., E.R.M.), University of Birmingham, Birmingham B15 2TT, United Kingdom; Department of Molecular Genetics (K.S., M.O., S.E.), Royal Devon and Exeter National Health Service Foundation Trust, Exeter EX2 5DW, United Kingdom; University of Exeter Medical School (S.E.), Exeter EX4 4PY, United Kingdom; Oxford Medical Genetics Laboratories (T.C.), Oxford University Hospitals National Health Service Trust, The Churchill Hospital, Oxford OX3 7LJ, United Kingdom; Section on Endocrinology and Genetics (P.X., C.A.S.) and Section on Molecular Dysmorphology (C.W.), *Eunice Kennedy Shriver* Institute of Child Health and Human Development, National Institutes of Health, Bethesda, Maryland 20892; Electron Microscopy Unit (L.M.), Department Histopathology, Charing Cross Hospital, Imperial College Healthcare National Health Service Trust, London W6 8RF, United Kingdom; Department of Clinical Genetics (A.K.), Great Ormond Street Hospital, London WC1N 1LE, United Kingdom; Departments of Oncology (N.F.) and Endocrinology (S.E.B.), University College London Hospitals, London WC1E 6BT, United Kingdom; Department of Diabetes and Endocrinology (D.M.), The Ipswich Hospital National Health Service Trust, Ipswich IP4 5PD, United Kingdom; Henry Wellcome Laboratories for Integrative Neuroscience and Endocrinology (S.L.), University of Bristol, Bristol BS1 3NY, United Kingdom; Department of Endocrinology (A.Ag., C.J.T.), Beaumont Hospital, Dublin 9, Ireland; Institute of Molecular and Experimental Medicine (A.R.), Cardiff University, Cardiff CF10 3US, United Kingdom; Department of Neurosurgery (J.G., M.P.), National Hospital for Neurology and Neurosurgery, London WC1N 3BG, United Kingdom; Servico de Endocrinologia e Metabologia (C.L.B.), Hospital de Clinicas, Universidade Federal do Parana, 80210 Curitiba, Brazil; Department of Endocrinology (P.D.), Post Graduate Institute of Medical Education and Research, Chandigarh 160012, India; Academic Endocrine Unit (R.V.T., M.S.), University of Oxford, Oxford OX1 3QX, United Kingdom; Christie Hospital National Health Service Foundation Trust (C.H.), Manchester M20 4BX, United Kingdom; Centre for Endocrinology and Diabetes (J.D.), Institute of Human Development, Faculty of Medical and Human Sciences, University of Manchester, Manchester M13 9PT, United Kingdom; Department of Endocrinology (J.D.), Manchester Royal Infirmary, Central Manchester University Hospitals National Health Service Foundation Trust, Manchester Academic Health Science Centre, Manchester M13 9WL, United Kingdom; Division of Genetics and Epidemiology (R.E.), The Institute of Cancer Research, London SW7 3RP, United Kingdom; Department of Histopathology (B.O., P.T., K.S.), University Hospitals Birmingham National Health Service Foundation Trust, Birmingham B29 6JD, United Kingdom; APHM Conception (A.B.), Laboratory of Molecular Biology, and Aix-Marseille Université, Centre National de la Recherche Scientifique, CRN2M-Unité Mixte de Recherche, 7286 Marseille, France; Departments of Endocrinology and Medicine (S.M.W., A.Au.), Hospital Sant Pau, IIB-Sant Pau, Centro de Investigación Biomédica en Red de Enfermedades Raras, Unit 747, Instituto de Salud Carlos III, 28029 Madrid, Spain; Universitat Autònoma de Barcelona, 08035 Barcelona, Spain; The JJR Macleod Centre for Diabetes, Endocrinology, and Metabolism (J.S.B.), Aberdeen Royal Infirmary, Aberdeen AB25 2ZB, Scotland, United Kingdom; Department of Endocrinology (C.P.), “Gr.T.Popa” University of Medicine and Pharmacy, 700115 Iasi, Romania; Endocrinology Research Centre (N.D.), Lomonosov Moscow State University, Moscow 115478, Russian Federation; Department of Internal Medicine (A.R.-O.), Federal University of Minas Gerais, 30330-120 Belo Horizonte, Brazil; Department of Medical Oncology and Cancer Genetics (I.T.G.), Castellon Provincial Hospital, 12002 Spain; Department of Pediatrics and Medical Genetics (G.Y., V.I.), University Multiprofile Hospital for Active Treatment “St. Marina,” 2010 Varna, Bulgaria; Department of Radiology (J.E.), St Bartholomew's Hospital, London E1 4NS, United Kingdom; Oxford Centre for Diabetes, Endocrinology and Metabolism (A.B.G.), University of Oxford, Oxford OX3 7LJ, United Kingdom; INSERM Unité 1028 (J.T.), Centre de Pathologie Est, Hospices Civils de Lyon, University of Lyon, 69622 Lyon, France; Department of Medical Genetics (E.R.M.), University of Cambridge, Cambridge CB2 1TN, United Kingdom; and Division of Brain Sciences (F.R.), Imperial College, London SW7 2AZ, United Kingdom

## Abstract

**Context:**

Pituitary adenomas and pheochromocytomas/paragangliomas (pheo/PGL) can occur in the same patient or in the same family. Coexistence of the two diseases could be due to either a common pathogenic mechanism or a coincidence.

**Objective:**

The objective of the investigation was to study the possible coexistence of pituitary adenoma and pheo/PGL.

**Design:**

Thirty-nine cases of sporadic or familial pheo/PGL and pituitary adenomas were investigated. Known pheo/PGL genes (*SDHA-D*, *SDHAF2*, *RET*, *VHL*, *TMEM127*, *MAX*, *FH*) and pituitary adenoma genes (*MEN1*, *AIP*, *CDKN1B*) were sequenced using next generation or Sanger sequencing. Loss of heterozygosity study and pathological studies were performed on the available tumor samples.

**Setting:**

The study was conducted at university hospitals.

**Patients:**

Thirty-nine patients with sporadic of familial pituitary adenoma and pheo/PGL participated in the study.

**Outcome:**

Outcomes included genetic screening and clinical characteristics.

**Results:**

Eleven germline mutations (five *SDHB*, one *SDHC*, one *SDHD,* two *VHL*, and two *MEN1*) and four variants of unknown significance (two *SDHA*, one *SDHB*, and one *SDHAF2*) were identified in the studied genes in our patient cohort. Tumor tissue analysis identified LOH at the *SDHB* locus in three pituitary adenomas and loss of heterozygosity at the *MEN1* locus in two pheochromocytomas. All the pituitary adenomas of patients affected by *SDHX* alterations have a unique histological feature not previously described in this context.

**Conclusions:**

Mutations in the genes known to cause pheo/PGL can rarely be associated with pituitary adenomas, whereas mutation in a gene predisposing to pituitary adenomas (*MEN1*) can be associated with pheo/PGL. Our findings suggest that genetic testing should be considered in all patients or families with the constellation of pheo/PGL and a pituitary adenoma.

The prevalence of symptomatic pituitary adenomas (PAs) in the general population is 1:1063 to 1:1282 (1, 2), whereas the prevalence of clinically diagnosed pheochromocytomas/paragangliomas (pheo/PGL) is 1:2500 to 1:6667 (3, 4). Although both are relatively rare diseases, PAs and pheo/PGL can sometimes occur in the same patient or in the same family. Coexistence of the two diseases could be due to pure coincidence, but it is possible that in some cases the two conditions share a common pathogenic mechanism. Since the first description of a patient with acromegaly and pheochromocytoma in 1952 (5), 70 cases have been published with this rare disease combination (Supplemental Tables 1–5). The simultaneous occurrence of these two tumor types might be explained by the following: 1) a pheo/PGL-related gene mutation, which, in addition to the pheo/PGL, also causes PA, as suggested for the *SDHX* mutation being involved in PA formation (6–8); 2) a mutation in a familial PA gene that also causes pheo/PGL; 3) a digenic disease, ie, two gene abnormalities are present in the same patient or family causing the two diseases; 4) a single, possibly novel, gene causing both diseases; 5) ectopic hypothalamic hormone-secreting adrenal tumors causing pituitary enlargement mimicking PA; or 6) the development of a pituitary adenoma and a pheo/PGL in the same patient or same family due to pure coincidence.

In the current study, we describe 39 cases of sporadic or familial pheo/PGL and PA in which a germline genetic analysis, loss of heterozygosity (LOH), and pathological studies were performed. Eleven germline mutations in five different genes (five *SDHB*, one *SDHC*, one *SDHD,* two *VHL*, and two *MEN1*) and four germline variants of unknown significance in three different genes (two *SDHA*, one *SDHB*, and one *SDHAF2*) were identified in the studied genes in our patient cohort. Tumor tissue analysis identified LOH at the *SDHB* locus in three pituitary adenomas and LOH at the *MEN1* locus in two pheochromocytomas. We have also identified a novel histological feature of *SDHX*-related PAs.

## Materials and Methods

### Patients

We collected clinical data, genomic DNA, and tumor tissue, when available, from 39 patients with pheo/PGL and PA in a sporadic (n = 19) or familial (n = 20) setting. Probands from 23 aryl hydrocarbon receptor interacting protein (*AIP*) mutation negative familial isolated PA (FIPA) families (defined as two or more subjects with pituitary adenomas but no syndromic features of other diseases such as multiple endocrine neoplasia (MEN)-1 or Carney complex) served as controls. Neurofibromatosis was ruled out based on clinical criteria according published guidelines (9). The study was approved by the local ethics committee and all subjects gave written informed consent.

### Genetic screening

#### Nucleic acid extraction

Genomic DNA was extracted from peripheral blood using a BACC2 DNA extraction kit (RPN-8502; GE Healthcare) according to the manufacturer's protocol. DNA extraction from formalin-fixed, paraffin-embedded pituitary or pheo/PGL tissue was performed using a QIAamp DNA FFPE tissue kit (QIAGEN). Representative tumor tissue was marked by a pathologist to avoid areas showing suboptimal preservation and contamination with normal tissue.

### Mutation testing

Sequence analysis of the *AIP* gene (NM_003977.2), MEN type 1 gene (*MEN1*; NM_130799.2), cyclin-dependent kinase inhibitor 1B gene (*CDKN1B*; coding region NM_004064.3, upstream open reading frame NM_004064.2) was performed using Sanger sequencing and multiplex ligation-dependent probe amplification (MLPA), as previously described (10–12). Genes implicated in pheo/PGL [MYC associated factor X (*MAX*; NM_002382.3), rearranged during transfection tyrosine kinase receptor gene (*RET*; NM_020975.4), succinate dehydrogenase subunit A (*SDHA*; NM_004168.2), succinate dehydrogenase complex assembly factor 2 (*SDHAF2*; NM_017841.2), succinate dehydrogenase subunit B (*SDHB*; NM_003000.2), succinate dehydrogenase subunit C (*SDHC*; NM_003001.3), succinate dehydrogenase subunit D (*SDHD*; NM_003002.2), transmembrane protein 127 (*TMEM127*; NM_017849.3), and von Hippel-Lindau gene (*VHL*; NM_000551.3)] were analyzed using a combination of next-generation sequencing, Sanger sequencing and MLPA, as previously described (13, 14). In addition, fumarate hydratase (NM_000143) was studied in a subset of patients. Tissue DNA analysis with PCR and sequencing was carried out according to standard protocols (Applied Biosystems). The sequences were analyzed using Mutation Surveyor (version 4.0.6; Softgenetics). In silico analysis of variants was performed using the Polyphen2 (http//:genetics.bwh.harvard.edu) and ALAMUT 2.2.0 (http://www.interactive-biosoftware.com/) softwares.

### Loss of heterozygosity analysis

Microsatellites D1S170 and D1S3669 for the *SDHB* locus were identified on the National Center for Biotechnology Information website (http://www.ncbi.nlm.nih.gov/) and the University of California, Santa Cruz Genome Browser website (http://genome.ucsc.edu/). Details of the microsatellites at the 11q13 locus (for *MEN1*) were previously described (15). Simple repeats were identified using the University of California, Santa Cruz website and designed accordingly for the specific region (15). The NCBI36/hg18 assembly of the human genome was used for the localization of the markers. Fragment analysis was carried out using standard protocols on an ABI 3730 (Applied Biosystems) and analyzed using GeneMarker (version 2.20; SoftGenetics). All primer sequences are available on request.

### Immunohistochemistry

Immunostaining for GHRH was performed using GHRH antibody 451–7 (Lyon, France), 1:2000 dilution, as previously described (16, 17). Pheochromocytomas of patients with the *MEN1* mutation were stained for menin using a rabbit polyclonal antimenin antibody (Abcam; ab2605, dilution 1:500), as previously described (18). Mouse pancreas showing islets and pheochromocytomas of patients without any known germline mutation were used as a positive control. SDHA and SDHB immunostaining was performed using a mouse monoclonal anti-SDHA antibody (2E3GC12FB2AE2, ab147159, dilution 1:200; Abcam) and a rabbit polyclonal anti-SDHB antibody (HPA002867, dilution 1:200; Sigma-Aldrich), as previously described (19). Further immunostaining was performed using the antimitochondrial antibody 113-1 recognizing a 60- to 65-kDa nonglycosylated membrane protein (Merck Millipore; dilution 1:150) and an antibody directed against the endoplasmic reticulum lectin 1 (ERLEC1; dilution 1:100; Novus Biological). Immunoreactions were performed using the automated Leica Bond III system. For antigen unmasking, EDTA at pH 8 was used for anti-113-1 and sodium citrate buffer (10 mM sodium citrate, 0.05% Tween 20, at pH 6) for anti-ERLEC1. The primary antibody binding was visualized with the SuperSentitive immunohistochemistry detection system from BioGenex. Sections were counterstained with Mayer's hemalum before being dehydrated and coverslipped.

### Statistical analysis

The statistical analysis was performed using StatsDirect software (Addison-Wesley-Longman). Normal distribution of the data was tested by the Shapiro-Wilk test. The Student *t* test was used to compare numerical variables. The χ^2^ or Fisher's exact tests were used to compare categorical variables. The results are reported as mean ± SD. Values of *P* < .05 were considered statistically significant.

## Results

### Clinical data

We identified 39 patients with sporadic (n = 19) or familial (n = 20 from eight families) pheo/PGL and PA. The gender distribution did not differ significantly (*P* = .6) in our cohort (18 males, 21 females) compared with the control group (12 males, 11 females). The mean age at diagnosis was 43.7 ± 18.2 years (mean ± SD) for PA and 47.2 ± 15.6 years for pheo/PGL (Supplemental Table 6). There was no significant difference in age of onset of PAs compared with the control group (35 ± 15.4; *P* = .08). In the PA-pheo/PGL cohort, comparing patients with and without mutation, no difference was identified in the age at diagnosis of the PA [mutation positive group (n = 12) 43.4 ± 18.9 y vs mutation negative group (n = 16) 44.8 ± 17.1 y, *P* = .8] or in the age of diagnosis of the pheo/PGL [mutation positive group (n = 15) 46.7 ± 14.3 y vs mutation negative group (n = 14) 48.4 ± 19.7 y, *P* = .8].

Nineteen patients had both pheo/PGL and PA, whereas a further 20 patients had pheo/PGL or PA in a setting detailed below. In two families (families 1 and 6), the proband had both PA and pheo/PGL, whereas other family members had either PA or pheo/PGL. In five families the pituitary and pheo/PGL tumors occurred in the same family but not in the same individual. One patient with a *VHL* mutation and a family history of clear-cell renal tumor and multiple hemangioblastomas had a PA presenting at 15 years (no typical VHL manifestations at this stage) (20). Two patients with *MEN1* mutations had a pheochromocytoma. One patient had acromegaly due to a GHRH-secreting pheochromocytoma (21).

Most PAs were lactotroph adenomas (n = 15), but somatotroph (n = 6), clinically nonfunctioning (n = 5, four of them showing positive FSH, LH or α-subunit immunostaining), and corticotroph (n = 1) adenomas were also seen. Twenty patients had macroadenomas and four patients had a microadenoma (for three patients PA size was not available). There was no significant difference (*P* = .8) in the pituitary adenoma size compared with the control group. Therapeutic modalities for pituitary disease included surgery, medical therapy (cabergoline or bromocriptine and somatostatin analogues), or radiotherapy. Twelve patients needed only one therapeutic intervention, and four patients needed two, three patients needed three, three patients needed four, and one patient needed five different therapeutic interventions (for three patients information on treatment modality was not available). One patient developed pituitary apoplexy.

Sixteen patients had pheochromocytomas and 14 patients had PGLs, of which 12 were head and neck PGLs and two were abdominal (retroperitoneal) PGLs.

### Genetic screening

Germline alterations were identified in *SDHA*, *SDHB*, *SDHC*, *SDHD*, *SDHAF2*, *VHL*, and *MEN1* genes in 19 patients with pheo/PGL and/or PAs. Fourteen of the 19 patients who harbored a genetic variant were index patients. All patients harbored one gene mutation except one patient, who had a *VHL* mutation and an *SDHA* variant of unknown significance. Twenty patients (including 10 harboring both pheo/PGL and PA) had no identifiable mutations in any of the genes tested (Table 1 and Supplemental Table 6). None of the patients in our cohort had *AIP* or *CDKN1B* mutations.

**Table 1.** Genes Tested in Pheo/PGL + Pituitary Adenoma Patient Cohort

Genes	Number of Patients With Sequence Variant	Sequence Variant	LOH in the Pituitary Adenoma	LOH in the Pheochromocytoma
*SDHA*	2 (2 variants)^a^	c.969C>T (p.Gly323Gly)^b^	No LOH	Not tested
		c.91C>T (p.Arg31Ter)		
*SDHB*	9 (8 mutations and 1 variant)	c.298T>C (p.Ser100Pro)	3 LOH	Tested and identified in 1 case
		c.587G>A (p.Cys196Tyr)		
		*SDHB* del exons 6–8		
		c.423 + 1G>A		
		c.770dupT (p.Asn258GlufsTer17)		
		Variant: c.80G>A (p.Arg27Gln)		
*SDHC*	2 (2 mutations)	c.380A>G (p.His127Arg)	NA	Not tested
*SDHD*	2 (2 mutations)	c.242C>T (p.Pro81Leu)	NA	Not tested
*SDHAF2*	1 (variant)	c.-52T>C	NA	Not tested
*VHL*	2^a^	c.340G>C (p.Gly114Arg)	No LOH^c^	Not tested
		c.589G>A (p.Asp197Asn)		
*MEN1*	2	c.1452delG (p.Thr557Ter)	Not tested	2 LOH
		c.783 + 1G>A		
*RET*	0			
*TMEM127*	0			
*MAX*	0			
*FH*	0			
*AIP*	0			
*CDKN1B*	0			

Abbreviations: FH, fumarate hydratase; NA, not available.

a One patient had two variants, a *VHL* and an *SDHA* variant.

b Further details are cited in Supplemental Table 6.

c LOH is not obligatory in VHL-related tumors [Banks RE, Tirukonda P, Taylor C, et al. Genetic and epigenetic analysis of von Hippel-Lindau (VHL) gene alterations and relationship with clinical variables in sporadic renal cancer. Cancer Res. 2006;66:2000–2011].

### *SDHX* mutation

**Figure 1.**
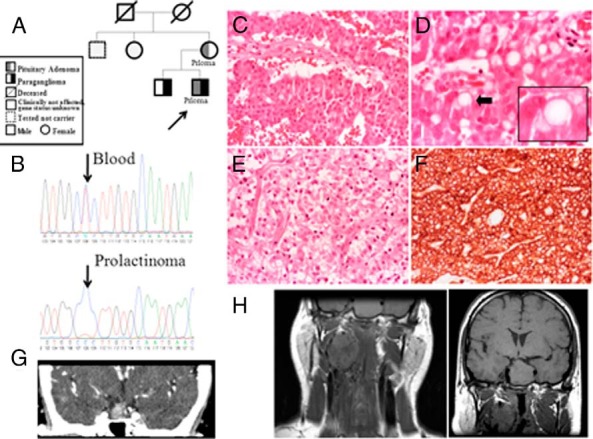
Pedigree (A) and LOH (B) at the *SDHB* locus in the pituitary adenoma of patient 1 in family 1 is shown. C, H&E staining of the pituitary adenoma of the proband (patient 1 in family 1) shows predominant trabecular architecture (×20). D, Vacuoles at times filling the entire cytoplasm characterize this case (arrow) (H&E, ×40). E, H&E staining (×20) of the pituitary adenoma of the proband's mother (patient 2 in family 1) also shows similar intracytoplasmic vacuoles. F, The immunoreaction with the anti-113-1 antibody (immunoperoxidase, ×20) shows the mitochondria content. G, MRI imaging of proband's mother's pituitary adenoma. H, MRI imaging of the proband's pituitary adenoma and glomus vagale tumor. MRI, magnetic resonance imaging.

**Figure 2.**
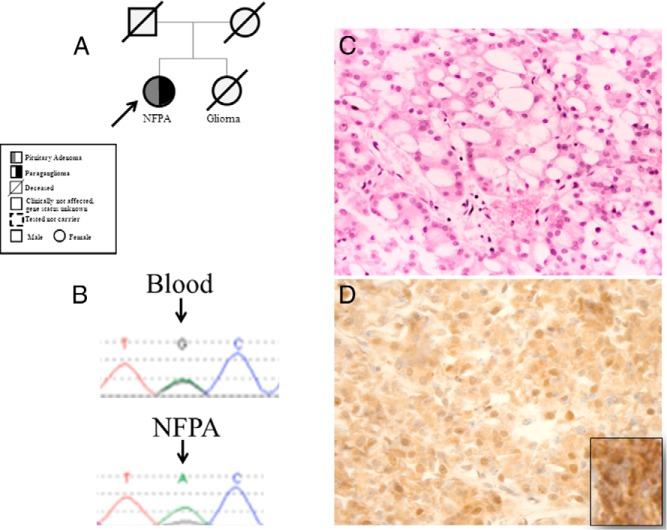
Pedigree (A) and LOH (B) at the *SDHB* locus in the pituitary adenoma of patient 4; the microsatellite upstream of the mutation has also shown to be lost. C, H&E-stained section (×20) of this adenoma shows prominent vacuolar changes in most neoplastic cells; the cytoplasm otherwise appears weakly eosinophilic. D, *SDHB* staining suggesting lack of strong granular staining of the pituitary adenoma of the proband (immunoperoxidase, ×20) (inset: positive SDHB staining as positive control in a paraganglioma).

**Figure 3.**
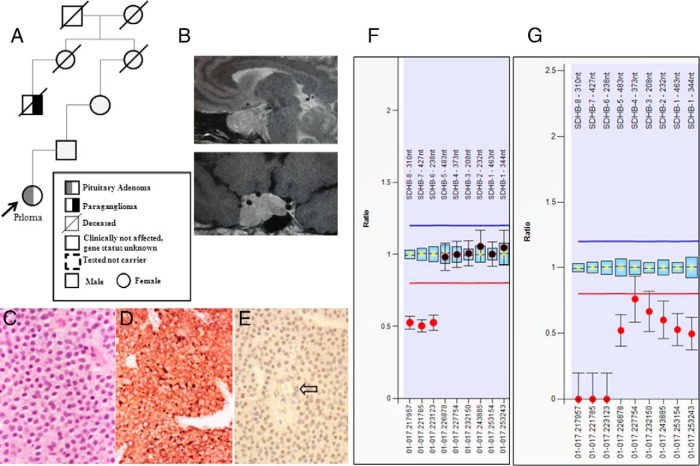
Pedigree (A) and sagittal and coronal magnetic resonance images of the pituitary adenoma (B) are shown. C, H&E-stained section (×20) shows that the tumor of patient 5 contains multiple vacuoles. D, The immunoreaction with the anti-113-1 antibody (immunoperoxidase, ×20) highlights the mitochondria content. E, SDHB immunostaining shows loss of expression in neoplastic cells, whereas endothelial cells (arrow) retain the expression (immunoperoxidase, ×20). Loss of the *SDHB* gene in germline and pituitary tumor tissue in patient 5. F, Germline DNA shows a deletion affecting MLPA *SDHB* probes 6–8 in DNA derived from leukocytes. G, In pituitary adenoma tissue, a complete loss of genetic material at the *SDHB* probes 6–8 area and heterozygous loss of *SDHB* probes 1–5.

We identified 11 kindreds (including 16 patients) with germline *SDHX* variants (Supplemental Table 6). Seven families had a pathogenic *SDH* mutation, whereas four had a variant of unknown significance. All patients with *SDHX* mutations/variants had a pituitary macroadenoma. In the pituitary adenomas, in which suitable sample was available, we identified the loss of the wild-type allele in the adenoma sample compared with the germline DNA (Figures 1–3). In particular, patient 5 was interesting in whom the germline mutation was a large deletion affecting exons 6–8 of the *SDHB* gene, whereas in the tumor sample the whole gene was deleted with no detectable exons 6–8 and a reduced amount of the other exons. We identified two *SDHA* variants of unknown significance. One of these (c.969C>T, p.Gly323Gly) was identified in a patient (patient 15) with a Wilms tumor (at the age of 1 y), retroperitoneal liposarcomas (32 and 40 y), a PGL in the retroperitoneum (50 y), a renal oncocytoma (50 y), and a nonfunctioning pituitary adenoma (NFPA; 53 y). His father had an NFPA operated at 44 years and again at 74 years. His mother (no known tumors) carried the c.969C<T variant. The other *SDHA* variant was identified in a patient with a *VHL* mutation and PA (patient 21). We have also identified an *SDHB* variant (c.80G>A p.Arg27Gln, patient 17) of unknown significance. We have tested the proband's pheochromocytoma and showed LOH at the *SDHB* locus; however, the SDHB staining of the pheochromocytoma did not show loss of SDHB expression. No pituitary tissue was available for testing in this family. An *SDHAF2* variant c.-52T>C was identified in a patient with somatotroph macroadenoma and head and neck PGL. The patient was not operated upon and therefore no tissue is available.

We identified two families with *SDH* mutations in which a family member with a PA did not carry the germline *SDHX* mutation: family 6 with two *SDHC* mutation-positive siblings had PA and/or PGL, whereas a first cousin had an NFPA but no *SDHC* mutation; and family 7 in whom the parent and child both had *SDHD* mutation-positive PGL and another child had a microprolactinoma but no *SDHD* mutation (Supplemental Figure 1). These cases are either phenocopies or could, theoretically, be explained by a digenic disease pattern in which the second disease-causing gene has not been identified.

### VHL mutation

An 18-year-old patient with a pathogenic *VHL* mutation [c.340G>C, a missense mutation affecting a surface amino-acid (22)], had an invasive GH- and prolactin (PRL)-positive PA as shown in Supplemental Table 6 and Supplemental Figure 2 (20).

### *MEN1* mutation

**Figure 4.**
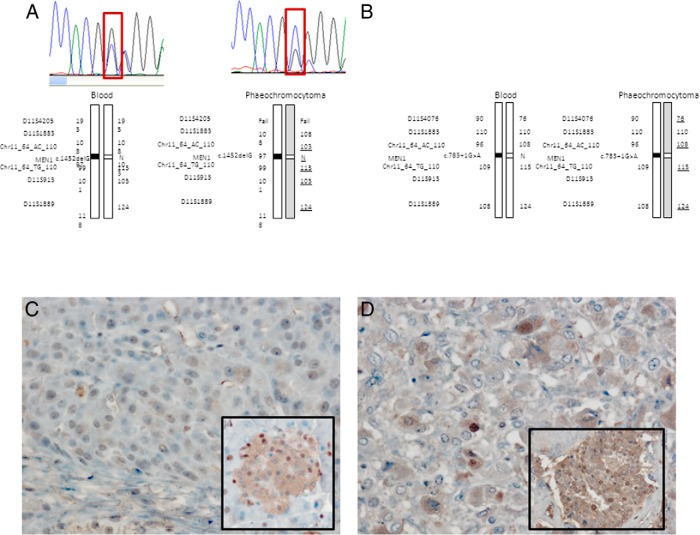
A, LOH analysis at the *MEN1* locus of the pheochromocytoma of patient 22 and patient 23 (B). Underlined microsatellite results identify markers that show a reduction in peak height in the pheochromocytoma sample compared with blood, indicating LOH but suggesting that some nontumoral tissue was also retained in the operated samples. C, Pheochromocytoma of patient 22 shows a loss of menin staining (inset: positive menin staining in mouse Langerhans islet). D, The menin staining of the pheochromocytoma of patient 23 shows some weakly positive staining nuclei (inset: positive menin staining in a sporadic pheochromocytoma used as a positive control).

We identified two patients (patients 22 and 23) with a germline *MEN1* mutation and pheochromocytoma, whereas all the other tested genes were normal (Supplemental Table 6). Both pheochromocytomas showed LOH in the *MEN1* gene, supporting, although not proving, the pathogenic role of *MEN1* in these tumors (see Figure 4, A and B). Although the association of pheo/PGLs and an MEN1-like syndrome has been described in the literature in 13 cases, in only four of these have *MEN1* mutations been identified (23–25), and none of them has been studied for LOH in the pheochromocytoma tissue.

### Control patients

We studied 23 *MEN1*-, *AIP*-, and *CDKN1B*-negative FIPA family probands without features of Carney complex or a personal or family history of pheo/PGL (Supplemental Table 7). We analyzed their DNA for all the pheo/PGL-related genes included in our panel to investigate the role of these genes in FIPA families. No pheo/PGL-related gene mutations were found in these families.

### Pathological features

The PAs of patients with *SDHX* mutations (patients 1 and 2 from family 1, patient 4, and patient 5) were characterized by intracytoplasmic vacuoles. The extent of vacuolization was not related to the histological type (prolactinoma or NFPA) of the tumor (Figures 1–3). The number of vacuolated cells varied from about 50% to 80% of the neoplastic cell population. Vacuoles ranged from small and multiple (Figure 3C) to large, occupying most of the cytoplasm and mimicking signet-ring cells (Figure 2C). None of the vacuoles indented the nucleus as commonly seen with accumulation of lipids. One case showed focal oncocytic changes identifiable on the hematoxylin and eosin (H&E)-stained sections. The histochemical stain periodic acid-Schiff (PAS)/diastase-resistant periodic acid of Schiff did not reveal any glycogen accumulation. Vacuoles were not seen in the PA of the patient with the germline *VHL* mutation (without *SDH* mutation) (Supplemental Figure 2). The SDHB staining of PAs with the *SDHB* mutation showed either a loss of expression of SDHB or a faint expression (Figures 2D and 3E).

Because *SDHX* mutations are known to alter mitochondrial function, immunostaining was performed for a mitochondrial membrane protein with the anti-113-1 antibody. This staining documented variable accumulation of mitochondria in *SDHX* mutation-positive PA cells. Some adenomas in particular showed increased immunostaining compared with the other cases (Figures 1F and 3D) in keeping with the focal oncocytic changes observed in the H&E-stained sections. Vacuoles did not appear to be rimmed by this protein, suggesting that vacuolization is not secondary to dilatation of mitochondria. To understand whether vacuoles were the result of swelling of the endoplasmic reticulum (ER), we immunostained our samples for the ER marker ERLEC1. None of the vacuoles was lined by this protein, indicating that they were not related to the ER (Supplemental Figure 3).

Menin staining of the pheochromocytoma samples of the patients with *MEN1* mutations showed either no menin-positive cells or weakly positive staining nuclei (Figure 4).

## Discussion

Syndromic presentation of PA and pheo/PGL is rare, and it is not part of the classical multiple endocrine tumor syndromes. This study describes, we believe, the largest cohort of patients with PAs and pheo/PGLs. Systematic testing of this population for alterations of the known pituitary and pheo/PGL-related genes suggest that *SDH* mutations play a pathogenic role in the development of PAs in some of these patients. Cases of other pheo/PGL genes associated with PA, *VHL* and *RET*, are exceptionally rare. On the other hand, the *MEN1* mutations can sometimes lead to pheo/PGLs, as suggested previously (23–25), and here we present supporting LOH and immunostaining findings. An endocrine rather than genetic association occurs when pheochromocytomas secrete hypothalamic-releasing hormones (GHRH or CRH) mimicking the PA and pheo/PGL syndrome, described previously in eight cases (Supplemental Table 2). Although in these cases only the adrenal gland harbors a tumor whereas the pituitary usually displays hyperplasia in response to the ectopic hormone secretion, this is a relevant clinical differential diagnostic scenario and should be kept in mind in patients with pituitary disease and pheo/PGLs. In approximately half of our cases, no germline abnormalities were seen, suggesting either the presence of other disease-causing genes or the coincidental occurrence of the pituitary and pheo/PGL tumors.

Because this is a multicentric study with a patient cohort from all over the world, with a heterogeneous genetic background, it is difficult to estimate whether the coincidence of these two tumors occurred randomly, or other, not-yet-specified genetic factors could be playing a role. Using the ranges of the available prevalence data for PAs and pheo/PGLs in the general population (1–4), the coincidental chance for the two diseases occurring in the same patient ranges between 1 in 2.5 million and 1 in 8.5 million subjects. In our single center (Barts), we reviewed 828 patients with pituitary tumors and 150 with pheo/PGL (26, 27). Assuming a maximum population frequency of pheo/PGL of 1 in 2500, we predict that 0.33 cases in a population-based series of 828 pituitary adenoma patients would have a pheo/PGL, whereas the actual frequency in patients seen at our center was 2 in 828 (*P* = .048; Fisher's exact test on single proportions). Likewise, assuming the maximum population frequency of PA of 1 in 1000, we expect 0.06 cases in a population-based series of 150 pheo/PGL patients would have a PA, whereas the actual frequency is 2 in 150 (*P* = .01). Both of these data sets suggest an increased incidence.

Of the six suggested explanations for the coexistence of PA and pheo/PGL that we outlined in the introductory text, we could confirm the following options: 1) a pheo/PGL-related gene causes PA, 2) a pituitary gene causes pheo/PGL, 5) ectopic hypothalamic hormone synthesis in a pheochromocytoma, and probably one or more families in our cohort match option, and 6) representing pure coincidence. Regarding option 3, we have not found any patients with mutations in two genes, such as a classical pheo/PGL and a pituitary tumor gene. In addition, we found LOH at the *SDH* locus in pituitary adenomas and at the *MEN1* locus in pheochromocytomas, suggesting, although not proving, that in these patients a single gene is responsible for both tumors. Exome or whole-genome sequencing studies in the future might find novel genes causing both diseases (option 4). In our cohort 19 patients (48%) had a germline alteration, among them 17 (43%) with a genetic variant in the pheo/PGL genes. Large studies showed that about one-third of pheo/PGL patients (most familial cases and 10%–20% of the sporadic cases) carry a germline mutation in *RET*, *VHL*, *NF1*, *SDHA*, *SDHB*, *SDHC*, *SDHD*, *SDHAF2*, *MAX*, or *TMEM127* genes (28, 29), suggesting that our cohort may have a slightly higher percentage of germline alterations.

The clinical features of the published cases of the association of pituitary disease and pheo/PGLs are summarized in the Supplemental Material (Supplemental Tables 1–5). More recently, three screening studies have been performed. One of them screened a group of patients (26 PGL patients and eight carriers) with a particular *SDHD* mutation due to a founder effect for the presence of a PA. One GH-secreting macroadenoma and three nonfunctioning microadenomas (suggested to be incidentalomas) were diagnosed in this patient cohort. No LOH was found at the *SDHD* locus in the GH-secreting PA (30). In the second study, 309 PAs were screened for *SDH* mutations and a macroprolactinoma with two different somatic *SDHA* mutations with normal sequence in the germline (31) was found. In the third study, screening has been performed in *SDHX*-mutated patients for nonpheo/PGL tumors. Two patients with *SDHD* mutations were found to have a PA, and in one of these cases, LOH at the *SDHD* locus was shown in the macroprolactinoma (32). Whether it is cost effective to measure prolactin in patients with pheo/PGLs needs to be studied further.

Summarizing our cases combined with the cases available in the literature (altogether 109 cases since 1952), we have tried to identify any particular features for each gene alteration for the tumor not classically associated with that gene. Twenty cases have a confirmed *SDHX* mutation with pituitary adenoma [(two *SDHA* (8, 31), eight *SDHB* (33, 34), two *SDHC* (35), and eight *SDHD* (30, 32, 36, 37)]. The patients with an *SDH* mutation had various PA types (Supplemental Tables 3 and 6): nine macroprolactinomas, three somatotroph adenomas, and five NFPAs have been described. In three cases the PA subtypes could not be classified. All the PAs were macroadenomas, except for three nonfunctioning microadenomas (possibly incidentalomas). The patients needed one to four therapeutic interventions. Five patients needed a single therapeutic intervention, five patients needed two, one patient needed three, and two patients needed four therapeutic interventions. Of the 109 patients, five patients had *RET* mutations (38–41); two cases with acromegaly, two cases with prolactinoma, and one NFPA (one macroadenoma and one microadenoma, and in three cases the adenoma size is not available). Four patients needed one therapeutic intervention (three surgeries and one medical treatment), whereas one patient needed medical therapy after transsphenoidal resection of the pituitary tumor. Two patients had a *VHL* mutation (20), one with a PRL and one with a GH- and PRL-secreting adenoma. Six patients had a confirmed *MEN1* mutation and pheo/PGL (23–25): five patients with pheochromocytoma and one head and neck PGL.

We have identified a novel feature of the PAs of patients harboring *SDHX* variants. The adenoma tissues show extensive vacuolization of cytoplasm with features reminiscent of signet-ring cells or physalipherous cells (42). The origin of vacuoles remains unclear. Lipid and glycogen accumulation was suggested in the literature, but none of the vacuoles indented the nucleus as commonly seen in cells with accumulation of lipids and the histochemical stain PAS/diastase-resistant periodic acid of Schiff did not reveal any glycogen accumulation. The vacuoles also do not resemble particle-rich cytoplasmic structures, described in epithelial neoplasms (43). Vacuolization of the nontumorous adenohypophyseal cells has been described in cases of fatal hypothermia in two separate studies (44, 45). Ishikawa et al (44) suggested that the vacuoles are different from dilated cisternae of rough ER and from distended Golgi apparatus, which are the result of castration or gonadal dysfunction and raised the possibility that they are lipid droplets due to metabolic dysfunction initiated by the hypothermia. Doberentz et al (45) also noted cytoplasmic vacuolation of the anterior pituitary cells in the case of hypothermia, and they suggested that this could be due to gradually developing tissue hypoxia. Oncocytic PAs have recently been identified to contain somatic mutations affecting mitochondrial respiratory chain complex I, but these tumors do not show the vacuolar changes we have identified in the *SDH*-related samples (46).

Inactivation of succinate dehydrogenase or VHL can lead to activation of the hypoxia inducible factor pathway and a pseudohypoxic state. Indeed, we have shown increased hypoxia inducible factor-1α in an *SDHD*-mutated case linked to pituitary adenoma (37). It is not known whether the vacuoles seen in the *SDH*-related tumors are due to the pseudohypoxic state, but we did not observe this phenomenon in the *VHL* mutation-related PA (Supplemental Figure 2).

Immunostaining for a mitochondrial membrane protein or for an ER marker did not prove that the vacuoles arise from these organelles. We attempted electron microscopy to identify the nature of the vacuoles, but this was inconclusive due to the poor preservation of formalin-fixed tissue recovered from paraffin (data not shown). These vacuoles were not specifically described in the studies of recently published *SDHX* mutations associated with PAs, but based on the available histological pictures, the presence of vacuoles cannot be ruled out (8, 31, 37). Vacuoles have been described in *SDHB* mutation-related renal carcinoma and were attributed to giant mitochondria (47), but the clear cytoplasm observed in these tumors can also represent glycogen or fat (48). Large cytoplasmic vacuoles suggested to be mitochondria based on electron microscopy have previously been described in PAs (49), possibly due to ischemia. Acidophil stem cell adenomas can also contain paranuclear vacuoles resulting from giant mitochondria (50).

The activity of certain mitochondrial enzymes involved in oxidative phosphorylation is decreased in cancer cells compared with normal tissue (51). Taking into account that succinate dehydrogenase enzymes, being part of the mitochondrial complex II, play an important role in mitochondrial function, mutations that affect the activity of these enzymes might have a role in mitochondria dysfunction (52). We believe that the vacuoles represent a hallmark of PA in patients with the *SDHX* variant, but their nature remains to be further investigated. In addition, further study of the metabolic pathways in *SDH*-related endocrine tumors are awaited.

Our study has several shortcomings. First of all, being a specialist pituitary and adrenal center with an interest in familial pituitary adenomas, our center might attract more unusual genetic conditions, therefore representing a higher prevalence of these cases. In a significant portion of the patients, tumor samples were not available, often due to the lack of surgical intervention; therefore, no appropriate material was available for LOH or to study in further detail the unusual histological phenotype in the PAs.

In summary, germline mutations were identified in the studied genes in 11 of 27 kindreds with the combination of pheo/PGL and PAs. LOH at the *SDHB* locus in the PA samples and LOH at the *MEN1* locus in the pheochromocytoma samples was demonstrated, suggesting, although not proving, the pathogenic role of these genes in these nonclassically disease-specific tissues. In addition, we noted intracytoplasmic vacuoles in PAs of patients affected by *SDH* mutations. Together with the single case reports available in the literature, this large cohort supports the hypothesis that in some families *SDH* mutations may have a role in PA formation and *MEN1* mutations may have a role in the development of pheochromocytoma. Whether screening for PAs in *SDHX* patients is warranted needs to be studied in the future, but our findings suggest that genetic testing for germline mutations in *SDHX* and *MEN1* should be considered in patients with the constellation of pheo/PGLs and PAs.
